# Torus mandibularis

**DOI:** 10.11604/pamj.2017.28.177.14038

**Published:** 2017-10-25

**Authors:** Pirabu Sakthivel, Chirom Amit Singh

**Affiliations:** 1 Department of Otorhinolaryngology & Head and Neck surgery, All India Institute of Medical Sciences, New Delhi, India

**Keywords:** Torus mandibularis, mandible, swelling

## Image in medicine

A 54-year-old man who presented to the clinic for a routine examination was found to have a hard swelling in the floor of mouth. The swelling was noticed at the age of ten years and had been gradually progressing over the years. However, there was no history of chewing difficulty, dysphagia, dysarthria, oral ulcers or sleep disturbances. On examination, a 4x4 cm bony swelling was noted arising from the lingual surface of mandible, with an intact overlying mucosa. The findings were typical of torus mandibularis, a benign bony outgrowth from the mandible which was also confirmed radiologically. About 80 to 90% of the lesions present as small, bilateral bony protrusions near the pre-molars and are incidental findings on routine oral examination. Despite the large size, our patient declined any treatment as the lesion was asymptomatic.

**Figure 1:**
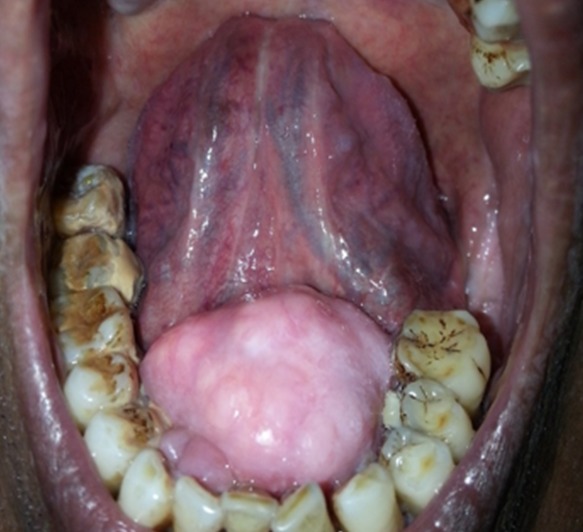
clinical image depicting the swelling on lingual surface of mandible with an intact overlying mucosa, the classical “Torus mandibularis”

